# Missing regulatory effects on complex traits: Contribution of distal variants

**DOI:** 10.1016/j.xgen.2025.100809

**Published:** 2025-03-12

**Authors:** Sool Lee, Hakhamanesh Mostafavi

**Affiliations:** 1 Center for Human Genetics and Genomics, New York University School of Medicine, New York, NY, USA; 2 Department of Population Health, New York University School of Medicine, New York, NY, USA

## Abstract

Most genetic effects on complex traits lie in non-coding regions, yet many show no regulatory activity in standard gene expression assays. In this issue of *Cell Genomics*, Arthur et al.^1^ add early development-like cell types and chromatin assays, showing that distal variants missed in expression assays partly explain this discrepancy.

## Teaser

Most genetic effects on complex traits lie in non-coding regions, yet many show no regulatory activity in standard gene expression assays. In this issue of *Cell Genomics*, Arthur et al. add early-development-like cell types and chromatin assays, showing that distal variants missed in expression assays partly explain this discrepancy.

## Main text

Genome-wide association studies (GWASs) are the standard approach for mapping genetic variants associated with human traits and diseases. By systematically analyzing genetic variation across the genome, GWASs have identified hundreds of thousands of variants associated with a broad range of phenotypes, including height, Alzheimer’s disease, and type 2 diabetes. Despite this success, extracting biological insights from GWASs remains a major challenge. One reason is that most GWAS variants (around 90%) are located in non-coding regions.^2^ These variants are presumed to act through regulatory effects, but for most, both their target genes and the contexts in which they affect gene expression remain unknown.

To address this, one approach is to test whether GWAS variants overlap with expression quantitative trait loci (eQTLs), which are variants that exhibit regulatory activity in gene expression assays. In practice, because both GWAS and eQTL loci implicate multiple variants due to linkage disequilibrium (LD), computational tools have been developed to test whether a given variant drives both GWAS and eQTL signals at a locus or whether they “colocalize.”^3^ However, despite the discovery of millions of eQTLs across various tissues and cell types, their overlap with GWAS variants is relatively small, leaving most GWAS loci unexplained.^4^ This has given rise to an ongoing puzzle, recently referred to as the “missing regulation” problem.^5^

There are two dominant explanations proposed for the problem of missing regulation: (1) missing GWAS variants are eQTLs that are context specific—such as being transiently active during development or induced upon stimulation—and are therefore overlooked by standard eQTL assays, which typically analyze postmortem bulk tissues,^6^ and (2) eQTL mapping is systematically biased away from trait-relevant genes, even when the right tissues and contexts are sampled, partly due to the effect of natural selection on human phenotypes.^7^ Moreover, variants with small regulatory effects, such as distal variants, are difficult to detect as eQTLs at current sample sizes but can still appear as GWAS hits if they regulate high-impact genes.^7^

**Figure 1:**
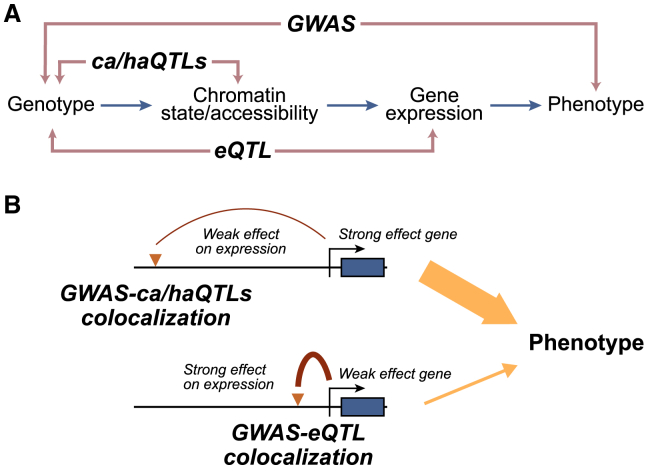
Complementary methodologies to study the regulatory function of genetic associations (A) Genetic variation can influence chromatin accessibility and state, gene expression, and ultimately phenotypic traits through a regulatory cascade. GWASs identify genotype-phenotype associations, while QTL mapping detects genetic associations with regulatory intermediates, including gene expression (eQTLs), chromatin accessibility (caQTLs), and histone acetylation (haQTLs). (B) Colocalization patterns of two GWAS hits (triangles) with similar phenotypic impact. One variant has a smaller effect on expression but regulates a higher-impact gene. eQTL signals are typically stronger for gene-proximal variants, potentially missing distal variants, which may be better captured by chromatin QTLs.

Arthur et al. set out to test these scenarios by expanding QTL mapping in two ways. First, they studied early development-like cell types, specifically induced pluripotent stem cells (iPSCs), iPSC-derived cardiovascular progenitor cells (CVPCs), and iPSC-derived pancreatic progenitor cells (PPCs), to assess how much of the missing regulation is due to unexplored contexts. Second, they complemented eQTLs with genetic effects on chromatin accessibility (caQTLs) and histone acetylation (haQTLs), a marker of active promoters and enhancers. While gene-proximal eQTLs typically have larger effects and are easier to detect, chromatin QTL discovery is likely independent of gene distance and may therefore improve colocalization detection at distal regulatory elements. Consistent with this expectation, chromatin QTLs showed weaker enrichment at promoters. However, it is important to note that if these assays had infinite statistical power, they should converge on the same set of variants, as they probe molecular intermediates along the same regulatory pathway (Figure 1A).

Across all development-like cell types and QTLs, Arthur et al. found colocalization for ∼10% of GWAS loci across 15 traits (540 out of 5,192). This is a relatively small number, likely due to the limited sample sizes they used for QTL mapping, on the order of a few hundred individuals. Nevertheless, colocalization patterns in these data provide insights into the missing regulation problem. Notably, incorporating chromatin QTLs led to a 2.3-fold increase in colocalization rates compared to eQTLs alone. Interestingly, GWAS-ca/haQTL colocalized variants were, on average, more distal than GWAS-eQTL colocalized variants. Furthermore, GWAS variants without any colocalization were farther from genes than colocalized GWAS variants. These observations support the model that some of the GWAS-eQTL gap arises from small regulatory effects of distal GWAS variants that are difficult to detect in gene expression assays.^7^ These variants may be better captured by chromatin QTLs (Figure 1B).

Strikingly, the majority of eQTLs identified in the early development-like samples (i.e., “EDev-like”) were also present in adult tissues; only ∼3% (2,299 out of 74,494 eQTL-gene pairs) were specific to EDev samples. These EDev-specific eQTLs exhibited smaller effects than shared eQTLs did, though this could arise if they tend to be more gene distal. More interestingly, EDev-specific eQTLs contributed minimally to colocalized GWAS signals (13 of 540). A similar pattern was observed in a previous study examining eQTLs during iPSC differentiation toward neural fates.^8^ Together, these findings suggest that broadly sampling additional developmental stages may not provide a straightforward solution to the missing regulation problem.

Lastly, the authors demonstrated the value of their multiomic QTLs in prioritizing variants within GWAS loci. Because nonfunctional variants can appear statistically associated with a trait due to LD, fine-mapping approaches have been developed to identify those most likely to have functional effects, defining them within a “credible set.” The authors show that integrating multiple lines of functional evidence—including colocalizing GWAS signals with different QTL types—can help narrow these credible sets to a smaller number of putative causal variants. Notably, most variants identified through this approach differed from those prioritized solely by statistical probability, consistent with recent evidence that the top statistical candidate is not always functional.^9^ While powerful, this strategy is limited to GWAS loci with sufficient functional data.

In conclusion, the work of Arthur et al. advances our understanding of the missing regulation problem, providing evidence consistent with both dominant explanations previously proposed. While it is essential to sample trait-relevant cell types and contexts, genetic effects on complex traits are possibly distributed across a broad range of regulatory contexts. As a result, studying any single context may not substantially increase overall colocalization, even though each newly explored context will likely contribute additional colocalized signals. Moreover, even in the most relevant cellular context, eQTL assays may be underpowered to detect distal regulatory variants. Chromatin QTLs may be particularly valuable in capturing these variants and could be complemented by computational approaches that predict chromatin architecture and regulatory landscapes from DNA sequence (e.g., Pampari et al.^10^). Ultimately, there seems to be no single solution to the missing regulation problem, and addressing it will likely require a multi-pronged approach.

## Declaration of interests

The authors declare no competing interests.
